# Evaluation of custom multiplex real - time RT - PCR in comparison to fast - track diagnostics respiratory 21 pathogens kit for detection of multiple respiratory viruses

**DOI:** 10.1186/s12985-016-0549-8

**Published:** 2016-06-06

**Authors:** Bharti Malhotra, M. Anjaneya Swamy, P. V. Janardhan Reddy, Neeraj Kumar, Jitendra Kumar Tiwari

**Affiliations:** Department of Microbiology & Immunology, Advanced Basic Sciences & Clinical Research Laboratory, (ICMR Grade – I Viral Diagnostics & Research Laboratory), Sawai Man Singh Medical College, Jawahar Lal Nehru Marg, Jaipur, 302 004 India

**Keywords:** Multiplex real time RT-PCR, Custom assay, FTD assay, Concordance

## Abstract

**Background:**

Severe acute respiratory infections in children can be fatal, rapid identification of the causative agent and timely treatment can be life saving. Multiplex real time RT-PCR helps in simultaneous detection of multiple viruses saving cost, time and labour. Commercially available multiplex real time RT-PCR kits are very expensive. Therefore the aim of the present study was to develop a cost effective multiplex real time RT-PCR for the detection of 18 respiratory viruses and compare it with an *in-vitro diagnostics* approved Fast Track Diagnostic Respiratory Pathogens 21 Kit (FTD).

**Methods:**

Nasopharyngeal aspirates and throat swabs were collected and processed for extraction of nucleic acid using an automated extraction system and multiplex real time RT-PCR was performed using the FTD kit and a custom assay on 356 samples.

**Results:**

Custom and FTD assays detected one or more respiratory viruses in 268 (75.29 %) and 262 (73.60 %) samples respectively. The concordance between the custom assay and the FTD assay was 100 % for HCoV OC43, HCoV 229E, HPIV-1, HPIV-2, HBoV, HPeV, Flu A, and *Influenza* A*(H1N1)pdm09* and 94.66 – 99.71 % for the remaining viruses; Flu B (99.71 %), HRV (99.71 %), HPIV-3 (98.87 %), HPIV-4 (99.43 %), HCoV NL63 (99.71 %), HMPV A/B (99.71 %), RSV A/B (94.66 %), EV (98.31 %), HCoV HKU1 (99.71 %), HAdV (99.71 %). Major discrepancy was observed for RSV A/B, which was over detected in 18 samples by the custom assay as compared to the FTD assay. The custom assay was much cheaper than the FTD assay and the time taken was only 29 min more.

**Conclusion:**

The custom primer and probe mix was found to be comparable to the FTD assay with good concordance but was much cheaper and the time taken for reporting was only 29 min more. The low cost custom multiplex RT-PCR can be a useful alternative to the costly FTD kit for rapid identification of viral aetiology in resource limited settings.

## Background

Severe acute respiratory infections (SARI) are one of the major causes of illness and death worldwide and are the third most common cause of death among children [1]. Acute respiratory infections (ARI) cause more deaths in children < 5 years with most cases reported from India (43 million), China (21 million), Pakistan (10 million), Bangladesh, Indonesia and Nigeria (56 million) [2]. Respiratory infections can be caused by many viruses, both DNA and RNA. These include the *Respiratory Syncytial Virus* (RSV), human *Parainfluenza* Virus (HPIV), *Influenza A* Virus (Flu A), *Influenza B* Virus (Flu B), human *Adenovirus* (HAdV), human *Coronavirus* (HCoV), human *Rhinovirus* (HRV), human *Metapneumovirus* (HMPV) and human *Bocavirus* (HBoV) [3]. A new wave of viral diagnosis was established with the development of Polymerase Chain Reaction (PCR) techniques in the 1990s [4]. PCR is more sensitive and rapid than conventional methods for detection of respiratory viruses. Different respiratory viruses present with similar signs and symptoms and can’t be differentiated symptomatically or clinically. Tests capable of rapid simultaneous identification of various viruses at the same time can help expedite initiation of appropriate therapy. Uniplex RT-PCR requires individual amplification of each virus under study which is expensive, time consuming and laborious [5]. To overcome this, multiplex real-time PCRs targeting the detection of multiple pathogens simultaneously have been developed commercially but they are very expensive. There is a need to develop cheaper systems for rapid simultaneous identification of various viruses. The present study compares custom real-time multiplex PCR primers and probes for the simultaneous detection of 18 respiratory viruses with an *in-vitro diagnostics* (IVD) approved fast track diagnostics (FTD) kit.

## Methods

### Patient inclusion criteria

Children with SARI, admitted in J. K. Lone Hospital, a pediatric hospital attached to Sawai Man Singh (SMS) Medical College Jaipur were enrolled in the study and tested for respiratory viruses with prior consent of the parent/guardian. Duration of the study was 27 months *i.e.* between September, 2012 to December, 2014. Children enrolled were ≤ 5 years of age, presenting with fever, cough, sore throat, nasal catarrh, shortness of breath, bronchiolitis, pneumonia, and wheezing.

### Patient exclusion criteria

**S**amples were not collected from patients with chronic respiratory ailments; non-consenting caregivers, with history of hospitalization in the preceding 14 days, not admitted in hospital and children aged > 5 years.

### Sample collection and transportation

A total of 356 nasopharyngeal aspirate and throat swab samples were collected from patients with SARI by a trained technician using a sterile nylon flocked swab and placed in viral transport medium (VTM), labelled and transported on ice at the earliest to Advanced research lab (ICMR Grade-1 Virology Lab) of SMS Medical college Jaipur for further processing and storage of the samples. The study was approved by the institutional ethics committee.

### Nucleic acid extraction

Viral nucleic acid from samples was extracted using an EasyMAG (Biomeurex) automated extractor according to the manufacturer’s instructions. Briefly, the extraction was done from 400 μl homogenised sample which was added to 1500 μl lysis buffer and was incubated for 10 min off board. The samples were loaded into the EasyMAG and 100 μl of magnetic silica was added to each sample and mixed well. Finally, the nucleic acid was eluted in a volume of 110 μl of which 50 μl was used for the FTD assay and 54 μl for the custom assay.

### Multiplex real -time RT-PCR (FTD ASSAY)

The multiplex real-time PCR FTD assay was performed on an ABI 7500 Fast instrument (Life Technologies, USA) as per the manufacturer’s instructions using an AgPath-ID ™ One-Step RT-PCR kit (Ambion) with the FTD Respiratory pathogens 21 kit (Fast Track Diagnosis, Luxembourg) for the detection of 18 viruses using five tubes containing primer and probe mix for different viruses; Tube-1 [*Influenza A* (Flu A), *Influenza A* subtype H1N1 (Pandemic H1N1), human *Rhinovirus* (HRV), *Influenza B* (Flu B)], Tube-2 [human *Coronaviruses* NL63 (HCoV-NL63), 229E (HCoV 229E), OC43 (HCoV-OC43), and HKU1 (HCoV HKU1)], Tube-3 [human *Parainfluenza* viruses, 2, 3, and 4 (HPIV- 2, 3 and 4) & IC], Tube-4 [human *Parainfluenza* viruses-1, Mycoplasma *pneumoniae* (M.pneu), human *Bocavirus* (HBoV), human *Metapneumovirus* (HMPV A/B)] and Tube-5 [*Respiratory Syncytial virus* (RSVA/B), human *Adenovirus* (HAdV), *Enterovirus* (EV), human *Parechovirus* (HPeV)]. The multiplex real time RT-PCR thermal profile for the FTD kit was as follows; 50 °C for 15 min, 95 °C for 10 min, 40 cycles of 95 °C for 8 s, 60 °C for 34 s, whereas the thermal profile for the custom assay was set at 50 °C for 30 min, 95 °C for 10 min, 45 cycles of 95 °C for 15 s, 55 °C for 30 s.

### Standardisation of uniplex real-time PCR (Custom assay)

Initial standardisations were done for uniplex real-time PCR using the FTD positive samples as controls. These positive samples included the following viruses namely, HRV, HAdV, Flu A, Pandemic H1N1, Flu B, HPIV-1, HPIV-2, HPIV-3, HPIV-4, RSVA/B, HMPV A/B, HPeV, EV, HCoV-OC43, HCoV-NL63, HCoV 229E, HCoV HKU1, (HBoV). AgPath (Ambion) one step RT-PCR master mix was used for the amplification and detection of viral nucleic acid. Briefly, each reaction was performed in a 20 μl volume which consisted of 12.5 μl of buffer, 1 μl of enzyme, 2 picomoles of each primer (corresponding to each virus), 2 picomoles of probe (corresponding to each virus) (synthesised by Life Technologies) (Table 1), 5 μl of extracted nucleic acid of the positive control/sample and made to a final volume of 20 μl with nuclease free water. The thermal cycling profile for the uniplex RT-PCR was 50 °C for 30 min (1 cycle), 95 °C for 10 min (1 cycle) followed by 90 °C for 15 s and 55 °C for 30 s (45 cycles). Specimens were considered positive when the Ct value was < 35.

**Table 1:** Custom primers and probes used for the detection of respiratory viruses

VIRUS	Froward primer (5’ - 3’)	Reverse primer (5’ - 3’)	Probe (5’ - 3’)^d^	References
Panel-1				
Flu B	GAGACACAATTGCCTACCTGCTT	TTCTTTCCCACCGAACCAAC	^a^FAM – AGAAGATGGAGAAGGCA AAG CAGA ACTAGC	Esposito et al., 2010 [17]
HCoV 229E	CAGTCAAATGGGCTGATGCA	AAAGGGCTATAAAGAGAATAAGGTATTCT	^b^VIC – CCCTGACGACCACGTTGTGGTTCA	Hammit et al., 2011 [6]
HCoV OC43	CGATGAGGCTATTCCGACTAGGT	CCTTCCTGAGCCTTCAATATAGTAACC	^c^NED-TCCGCCTGGCACGGTACTCCCT	Hammit et al., 2011 [6]
Panel-2				
HPIV-4	CAGAYAACATCAATCGCCTTACAAA	TGTACCTATGACTGCCCCAAARA	^a^FAM – CCMATCACAAGCTCAGAAATYCAAAGTCGT	Hammit et al., 2011 [6]
HPIV-1	GTGATTTAAACCCGGTAATTTCTCA	CCTTGTTCCTGCAGCTATTACAGA	^b^VIC- ACCTATGACATCAACGAC	Hammit et al., 2011 [6]
HPIV-3	CCAGGGATATAYTAYAAAGGCAAAA	CCGGGRCACCCAGTTGTG	^c^NED – TGGRTGTTCAAGACCTCCATAYCCGAGAAA	Hammit et al., 2011 [6]
Panel-3				
Influenza A(H1N1) pdm09	GTGCTATAAACACCAGCCTYCCA	CGGGATATTCCTTAATCCTGTRGC	^a^FAM - CAGAATATACATCCRGTCACAATTGGARAA	WHO, 2009 [22]
HRV	TGGACAGGGTGTGAAGAGC	CAAAGTAGTCGGTCCCATCC	^b^VIC - TCCTCCGGCCCCTGAATG	Hammit et al., 2011 [6]
HPIV-2	ATGAAAACCATTTACCTAAGTGATGGA	CCTCCYGGTATRGCAGTGACTGAAC	^c^NED - TCAATCGCAAAAGC	Hammit et al., 2011 [6]
Panel-4				
RSV A/B	GGAAACATACGTGAACAAGCTTCA	RSV-A: CATCGTCTTTTTCTAAGACATTGTATTGA	^a^FAM – TGTGTATGTGGAGCCTT	Kwofie et al., 2012 [10]
		RSV-B: TCATCATCTTTTTCTAGAACATTGTACTGA		
HCoV NL63	ACGTACTTCTATTATGAAGCATGATATTAA	AGCAGATCTAATGTTATACTTAAAACTACG	^b^VIC –ATTGCCAAGGCTCCTAAACGTACAGGTGTT	Hammit et al., 2011 [6]
HCoV HKU1	AGTTCCCATTGCTTTCGGAGTA	CCGGCTGTGTCTATACCAATATCC	^c^NED - CCCCTTCTGAAGCAA	Cui et al., 2011 [11]
Panel-5				
EV	CCCTGAATGCGGCTAATCC	ATTGTCACCATAAGCAGCCA	^a^FAM- AACCGACTACTTTGGGTGTCCGTGTTTC	Wolffs et al., 2011 [23]
HPeV	GTAACASWWGCCTCTGGGSCCAAAAG	GGCCCCWGRTCAGATCCAYAGT	^b^VIC- CCTRYGGGTACCTYCWGGGCATCCTTC	Nix et al., 2008 [12]
HBoV	TGCAGACAACGCYTAGTTGTTT	CTGTCCCGCCCAAGATACA	^c^NED – CCAGGATTGGGTGGAACCTGCAAA	Sanghvi et al., 2012 [22]
Panel-6				
Flu A	GACCRATCCTGTCACCTCTGAC	AGGGCATTYTGGACAAAKCGTCTA	^a^FAM – TGCAGTCCTCGCTCACTGGGCACG	WHO, 2009 [22]
HAdV	GCCCCAGTGGTCTTACATGCACATC	GCCACGGTGGGGTTTCTAAACTT	^b^VIC – TGCACCAGACCCGGGCTCAGGTACTCCGA	Hammit et al., 2011 [6]
HMPV A/B	CATCAGGTAATATCCCACAAAATCAG	GTGAATATTAAGGCACCTACACATAATAARA	^c^NED - TCAGCACCAGACACAC	Sanghvi et al., 2012 [13]

NOTE: The lower limit for the detection of HBoV- 1 DNA copy/ml, HMPV- 30 Rna copies/ml, HPeV- 10^3^ (cell culture infective dose) CCID_50_ - 10^4^ CCID_50_, RSV A/B- 2×10^4^copies/μl, HCoV HKU1- 5×10^3^ copies/ml, Flu B- 2.2 Log_10_ (viral particles) VP/ml, and Influenza A(H1N1)pdm09 - 2×10^1^ to 2×copies/ml

^a^FAM - Detection wavelength - 518 nm; ^b^VIC detection wavelength - 554 nm; ^c^NED detection wavelength 575 nm

^d^All the probes were having (non fluorescence quencher) NFQ as quencher at 3’ end

### Standardisation of multiplex real-time RT-PCR (Custom assay)

Uniplex PCR was followed by multiplex real-time RT-PCR standardisation. In multiplex PCR, each reaction was targeted for the simultaneous detection of three different viruses in a single reaction tube. Each reaction mix consisted of respective forward and reverse primers, and probes each labelled with a different fluorescent dye (FAM, VIC and NED) specific to each of the three viruses. Tube-1 [*Influenza B* (Flu B), human *Coronaviruses*229E (HCoV 229E), OC43 (HCoV-OC43)], Tube-2 [human *Parainfluenza* viruses, 1, 3, and 4 (HPIV- 1, 3 and 4)], Tube-3 [*Influenza A* subtype H1N1 (Pandemic H1N1), human *Rhinovirus* (HRV), human *Parainfluenza* viruses-1 (HPIV-1)], Tube-4 [*Respiratory Syncytial virus* (RSVA/B), human *Coronaviruses* NL63 (HCoV-NL63), HKU1 (HCoV HKU1)], Tube-5 [*Enterovirus* (EV), human *Parechovirus* (HPeV), human *Bocavirus* (HBoV)], Tube-6 [*Influenza A* (Flu A), human *Adenovirus* (HAdV), human *Metapneumovirus* (HMPV A/B)]. FTD positive samples were considered as standard. AgPAth (Ambion) one step RT-PCR master mix was used for the detection of respective viral nucleic acids as mentioned above in a reaction volume of 25 μl but with 9 μl of nucleic acid, 4 picomoles of each primer and 2 picomoles of probe. The thermal profile for the multiplex real-time PCR was as described above. Specimens were considered positive when the Ct value was < 35. After standardisation of multiplex PCR the same protocol was used for screening patient samples. Rnase P was used as an internal control in a separate uniplex RT PCR assay. Samples negative for Rnase P were not included in the study.

## Results

A total of 356 samples were tested by both assays. Custom and FTD assays detected one or more respiratory viruses in 268 (75.29 %) and 262 (73.60 %) samples respectively (Table 2).

**Table 2:** Single and multiple infections detected by custom assay as compared to FTD assay

Number of viruses detected	Custom assay	FTD assay
0	88 (24.71 %)	94 (26.40 %)
1	197 (55.33 %)	203 (57.02 %)
2	65 (18.25 %)	55 (15.44 %)
3	6 (1.68 %)	4 (1.12 %)

No significant differences were seen in the number of samples positive for each virus by the custom assay as compared to the FTD assay except with RSV A/B which was over detected in 18 samples and one sample being under detected by the custom assay as compared to the FTD assay. Further, to completely assess the results of these 18 discordant RSVA/B samples, testing was repeated using RSV A and RSV B specific primer and probe mix in uniplex real time RT- PCR as published previously [6]. All 18 samples were found to be positive for RSV B (Table 3).

**Table 3:** Typing results of RSV A & RSV B for samples positive by custom assay

RSV A	RSV B	RSV A & B (co-infections)	Total
0/18	18/18	0/18	18/18

One hundred percent concordance was observed between the custom assay and the FTD assay for eight viruses; HCoV OC43, HCoV 229E, HPIV-1, HPIV-2, HBoV, HPeV, Flu A, and *Influenza* A*(H1N1)pdm09* while it varied from 94.66 to 99.71 % for the remaining ten viruses; Flu B, HRV, HPIV-3, HPIV-4, HCoV NL63, HMPV A/B, RSV A/B, EV, HCoV HKU1, HAdV. (Table 4).

**Table 4:** Concordance between custom and FTD assay

Virus	FTD (+) Custom (+)	FTD (+) Custom (-)	Custom (+) FTD (-)	Concordance between custom assay and FTD assay in %
HCoV OC43	7	0	0	100.0
HCoV 229E	3	0	0	100.0
Flu B	12	0	1	99.71
HPIV-1	6	0	0	100.0
HPIV-3	22	1	3	98.87
HPIV-4	5	1	1	99.43
HPIV-2	5	0	0	100.0
HCoV NL63	2	1	0	99.71
HMPV A/B	42	1	0	99.71
RSV A/B	66	1	18	94.66
HBoV	16	0	0	100.0
HPeV	1	0	0	100.0
HRV	65	0	1	99.71
EV	8	2	4	98.31
HCoV HKU1	2	1	0	99.71
HAdV	26	0	1	99.71
Flu A	2	0	0	100.0
*Influenza* A(H1N1)pdm09	27	0	0	100.0

Low concordance was observed between the two assays for RSV A/B (94.66 %) and EV (98.31 %).

The discordant results of the custom assay were seen in 19 co-infection samples, 13 single infection samples and four negative samples as compared to the FTD assay, and the discordance was predominant in the co-infected samples as compared to single infection samples (Table 5).

**Table 5:** Discordant results of custom and FTD assays

Custom assay	FTD assay	Number of samples
RSV A/B	NEGATIVE	07
HBoV + RSV A/B	HBoV	02
HRV + RSVA/B	HRV	02
EV + RSV A/B	EV	01
HMPVA/B + RSV A/B + Flu B	HMPVA/B + Flu B	01
*Influenza* A(H1N1)pdm09 + RSVA/B + HBoV	*Influenza* A(H1N1)pdm09 + HBoV	01
HMPVA/B + RSV A/B	HMPVA/B	01
Flu B + RSV A/B	Flu B	01
HPIV-3 + RSV A/B	HPIV-3	01
HPIV-3 + HRV	HRV	01
HBoV + HPIV-3	HBoV	01
^a^HPIV-4 + HRV	^a^HPIV-3 + HRV	01
EV	NEGATIVE	02
NEGATIVE	EV	01
HBoV	HBoV + EV	01
HCoV OC43	HCoV OC43+ HCoV HKU1	01
HRV + HAdV	HRV	01
HPIV-2 + Flu B	HPIV-2	01
HPIV-3	NEGATIVE	01
NEGATIVE	HPIV-4	01
NEGATIVE	HCoV NL63	01
HPIV-3	HPIV-3 + HMPVA/B	01
*Influenza* A(H1N1)pdm09 + EV	*Influenza* A(H1N1)pdm09	01
HRV + RSVA/B	HRV	01
NEGATIVE	RSV A/B	01
HRV + EV	HRV	01

^a^This sample is counted as variation of co-infection on both the sides

Comparisons between the custom assay and the FTD assay were made based on the different parameters listed in Table 6. Most of the findings between the custom assay and the FTD assay were similar except for the cost incurred for screening 18 respiratory viruses. In this regard, the custom assay was found to be more economical than the commercial FTD assay.

**Table 6:** Comparison of custom and FTD assay in regard to sample testing

	Custom assay	FTD assay
Viral Pathogens	Flu A, *Influenza*	Flu A, *Influenza*
	A(H1N1)pdm09, Flu B	A(H1N1)pdm09, Flu B
	HPIV - 1, 2, 3, 4	HPIV - 1, 2, 3, 4
	HCoV OC43, 229E, NL63,	HCoV OC43, 229E, NL63,
	HKU1,	HKU1,
	RSV A/B	RSV A/B
	HMPV A/B	HMPV A/B
	EV	EV
	HPeV	HPeV
	HRV	HRV
	HAdV	HAdV
	HBoV	HBoV
	--	M. pneu
Sample volume	200 μl	200 μl
Elution volume	55 μl	55 μl
PCR volume	25 μl	25 μl
Principle of detection	Fluroscence probe based	Fluroscence probe based
Hands on time	50 min	50 min
Time to result	3.5 h	3.01 h
Total cost per sample	INR 1500	INR 4300
Ease of Handling	+	+
Equipment	*NucliSENS EasyMAG, ABI 7500 DX Fast*	*NucliSENS EasyMAG, ABI 7500 DX Fast*
No. of PCR tubes used per sample	6	5
Primers & probes	Custom assay (ABI)	FTD
Number of targets/tube	3	4
PCR reaction volumes		
2X RT-PCR buffer	12.5 μl	12.5 μl
25X RT-PCR enzyme	1.0 μl	1.0 μl
Primer probe mix/tube	1.8 μl	1.5 μl
Nucleic acid	9 μl	10 μl
Nuclease free water	0.7 μl	--

## Discussion

The present study was performed to compare a custom multiplex assay and an FTD multiplex assay by testing of 356 respiratory samples obtained from children with SARI admitted in J K lone paediatric hospital Jaipur.

In the present study, the concordance between the custom assay and the FTD assay was found to be 100 % for Flu A, *Influenza* A(H1N1) pdm09, HCoV OC43, HCoV 229E, HPIV-1, HPIV-2, HBoV, and HPeV. Similarly Chen et al., [7] reported a concordance of 99.60 % for Flu A and *Influenza* A(H1N1) pdm09 when comparing a multiplex PCR assay with a uniplex assay.

The concordance between the two assays varied from 94.66 to 99.71 % for the remaining ten viruses; Flu B (99.71 %), HPIV-3 (99.71 %), HPIV-4 (99.43 %), HCoV NL63 (99.71 %), HMPV A/B (99.71 %), RSV A/B (94.66 %), HCoV HKU1 (99.71 %), HAdV (99.71 %), HRV (99.71 %), EV (98.31 %). Similar findings have been observed in earlier studies for Flu B (98.25 to 99.42 %), HPIV-3 (96.53 to 99.30 %), HPIV-4 (97.10 %), HCoV NL63 (95.95 to 100.0 %), HMPV A/B (99.65 to 100.0 %), RSV A/B (93.06 to 98.60 %), HCoV HKU1 (98.84 to 100.0 %), HAdV (97.20 to 100.0 %) [8, 9]. Concordance for EV in the present study was different from an earlier study (93.00 %) [8]. The difference in concordance obtained in different studies may be due to the different primer binding regions or may be due to different methodologies employed by various studies. The number of samples positive for HCoV 229E, HPIV-4, HPIV-2, HCoV NL63, HPeV, HCoV HKU1, Flu A, were ≤ 5 in the present study. Studies based on larger numbers of samples are required to assess the concordance of these viruses more thoroughly.

The limit of detection for some of the viruses in the custom assay (Table 7) ranged from 1 DNA copy/ml to 2×10^4^ copies/ml [7, 10–14]. The detection limit of the FTD assay for different viruses was 10^2^ copies/ml for FluA, HPIV-2, HMPV and HCoV OC43; 10^3^ copies/ml for FluB, HCoV HKU1, HPIV-1, HBoV, HPIV-3, HCoV NL63, RSV, HAdV, EV, and HPeV; and 10^4^ copies/ml for HRV, HCoV 229E and HPIV-4 [15].

**Table 7:** Detection limits of different respiratory viruses by custom assay and FTD kit

Name of the virus	Custom assay detection limit	FTD assay detection limit
HBoV	1 DNA copy/ml [13]	10^3^ copies/ml
HMPV	30 RNA copies/ml [13]	10^2^ copies/ml
HPeV	10^3^ CCID_50_ - 10^4^ (CCID_50_ [12]	10^3^ copies/ml
RSV A/B	2 × 10^4^ copies/ml [10]	10^3^ copies/ml
HCoV HKU1	5 × 10^3^copies/ml [11]	10^3^ copies/ml
Flu B	2.2 Log_10_ VP/ml [14]	10^3^ copies/ml
Influenza A(H1N1)pdm09	2 × 10^1^ to 2 × 10^2^ copies/ml [7]	10^3^ copies/ml

In the present study RSV A/B was the most predominant virus detected by both the custom and FTD assays with positivity in 84 (23.60 %) and 67 (18.82 %) samples respectively and concordance of 94.66 %. This finding is different when compared with other studies [8, 16] where comparisons were made between multiplex PCRs in which RSV was the second most predominant virus detected [16].

The custom primer and probes used for *Influenza* A(H1N1) pdm09, RSV A/B, Flu B, HMPV A/B, HBoV, HRV, HPIV-1-4, HAdV and HCoVs showed a positivity of 7.58, 23.60, 3.65, 11.80, 4.49, 18.54, 11.79, 7.58 and 3.93 % respectively for each virus in the present study in comparison to a positivity of 18.39 % [7], 14.1 % [10], 13.3 % [17], 2.9 % [13], 0.5–4.5 % [13, 18, 19], 20.78 % [6], 8.62 % [6], 3.5 % [20], and 4.70 % [6] respectively in earlier studies where the same primer and probes were used. HBoV was mostly associated with co-infections in the present study in both assays. This is consistent with an earlier study [16]

The major discrepancy in the present study was found with RSV A/B. The discrepancy in 18 samples which were over detected by the custom assay was resolved by RSV A and RSV B typing. The RSV typing results for the discrepant samples showed that all 18 samples were RSV B. Further all samples positive for RSV A/B by the FTD assay were also subjected to RSV typing which indicated RSV A in 13 (19.40 %) samples, RSV B in 50 (74.63 %) samples and RSV A & RSV B dual infections in 4 (5.97 %) samples

During the process of standardisation of the custom assay 3 μl of viral nucleic acid (positive control) was used for each virus including 4 picomoles of primers and 2 picomoles of probes. Each panel consisted of 3 viruses. In total 9 μl of viral nucleic acid was used for each panel. While the FTD assay used 10ul of nucleic acid in each tube with primers and probes for 4 viruses, the concentration of primer and probe was not disclosed by FTD. In total 4 μl more of viral nucleic acid was used in the custom assay compared to the FTD assay which may have increased the sensitivity/detection of different viruses in the custom assay.

Initially during the process of standardisation of the custom assay, different primer and probe concentrations were tried and the PCR was run for 45 cycles as per the protocol followed by various authors. Although data was analysed using PCRs run for 35 and 40 cycles, best results were achieved using a Ct value of 35 for both the FTD assay and the custom assay. Accordingly, a Ct value of <35 was considered as positive for both assays as per the FTD kit. With the custom assay being run for 40 cycles this reduces the custom assay run time by 8 min, thereby making it only 21 min longer than the FTD assay.

Comparisons were made between various aspects of the custom and the FTD assays (Table 6). No major differences were observed between the two assays except in the cost incurred for both assays. Similar comparisons were also done in an earlier study [21] where three multiplex PCRs were compared. The turn-around time of the custom assay was 29 min more as compared to the FTD assay. But both the assays reported the results on the same day. The excess time of 29 min taken by the custom assay as compared to the FTD assay may not greatly interfere with the treatment process. However, the custom assay was much more economical costing INR 1500/- per sample for screening 18 respiratory viruses compared to the commercial FTD assay which was expensive costing INR 4300/- per sample. This assay may prove to be highly cost effective in resource limited settings like ours. However the limitation of our study was that some of the viruses showed low positivity as a result it is difficult to assess the concordance accurately. Larger numbers of positive samples need to be tested to evaluate the concordance of these less prevalent viruses.

## Conclusion

This study reported a high prevalence of respiratory viruses in children ≤ 5 years using a custom assay and an FTD assay. Good concordance was observed for all the viruses between both assays except for RSVA/B. However larger numbers of positive samples need to be tested for thorough evaluation of less prevalent viruses. The custom primer and probe mix was much more economical than the commercial FTD kit. Our study suggests that this custom multiplex real-time RT-PCR can be used for simultaneous and rapid detection of multiple viruses in resource limited settings. This will help prevent unnecessary use of antibiotics and permit timely initiation of supportive therapy/antiviral drugs if available.

## Abbreviations

ARI, acute respiratory infections; CCID, cell culture infective dose; EV, *enterovirus*; Flu A, *influenza A*; Flu B, *influenza B*; FTD, fast track diagnostic; HAdV, human *adenovirus*; HBoV, human *bocavirus*; HCoV 229E, human *coronaviruses* 229E; HCoV HKU1, human *coronaviruses* HKU1; HCoV-NL63, human *coronaviruses* NL63; HCoV-OC43, human *coronaviruses* OC43; HMPV A/B, human *metapneumovirus;* HPeV, human *parechovirus*; HPIV- 1, 2, 3 and 4, human *parainfluenza* viruses 1, 2, 3, and 4; HRV, human *rhinovirus*; IVD, *in-vitro* diagnostics; M.pneu, mycoplasma pneumoniae; NFQ, non fluorescence quencher; Pandemic H1N1, *influenza A* subtype H1N1; PCR, polymerase chain reaction; RSVA/B, *respiratory syncytial virus*; SARI, sever acute respiratory infections; SMS, Sawai Man Singh; VP, viral particles; VTM, viral transport medium
